# The hedgehog pathway and ocular developmental anomalies

**DOI:** 10.1007/s00439-018-1918-8

**Published:** 2018-08-02

**Authors:** Florencia Cavodeassi, Sophie Creuzet, Heather C. Etchevers

**Affiliations:** 1 Institute for Medical and Biomedical Education, St. George´s University of London, Cranmer Terrace, London, SW17 0RE UK; 2 Institut des Neurosciences Paris-Saclay (Neuro-PSI), UMR 9197, CNRS, Université Paris-Sud, 1 Avenue de la Terrasse, 91198 Gif-sur-Yvette Cedex, France; 3 Aix-Marseille Univ, Marseille Medical Genetics (MMG), INSERM, Faculté de Médecine, 27 boulevard Jean Moulin, 13005 Marseille, France

## Abstract

Mutations in effectors of the hedgehog signaling pathway are responsible for a wide variety of ocular developmental anomalies. These range from massive malformations of the brain and ocular primordia, not always compatible with postnatal life, to subtle but damaging functional effects on specific eye components. This review will concentrate on the effects and effectors of the major vertebrate hedgehog ligand for eye and brain formation, Sonic hedgehog (*SHH*), in tissues that constitute the eye directly and also in those tissues that exert indirect influence on eye formation. After a brief overview of human eye development, the many roles of the SHH signaling pathway during both early and later morphogenetic processes in the brain and then eye and periocular primordia will be evoked. Some of the unique molecular biology of this pathway in vertebrates, particularly ciliary signal transduction, will also be broached within this developmental cellular context.

## Introduction

The hedgehog signaling pathway was named after the denticle phenotype of larval Drosophila fruit fly mutants, in which the alternating pattern of abdominal bristles and naked cuticle was replaced by a stubby and uniformly fuzzy appearance. The first description presciently grouped the “hedgehog” (*hh*) and “cubitus interruptus” (*Ci*) mutants among those affecting this segmental polarity pattern (Nüsslein-Volhard and Wieschaus 1980). As the corresponding genes were identified and characterized, it turned out that in all vertebrates including humans, *hh* ligand and *Ci* transcription factor orthologs, as well as dozens of intermediate effectors, are functionally conserved in many ways and have diversified in others. Hedgehog signaling pathways are now known to regulate many fundamental cellular processes of patterning, cell identity, and environmental responsiveness in all Eumetazoa (Matus et al. 2008; Sigg et al. 2017). They have been doing so over the last 940 million years (Nikoh et al. 1997).

The hedgehog pathway, impinging on similarly conserved/diversified transcriptional gene regulation networks, has been co-opted during the evolution of numerous complex organ systems in the vertebrate clade, particularly the eye (Nilsson 2004; Meulemans and Bronner-Fraser 2005). Non-coding, cis-regulatory elements control when and where not only the ligands, but also the effectors and potential downstream targets may be deployed (Jeong et al. 2006; Cvekl and Duncan 2007); as such, these may also account for some of the complex genetics behind human eye malformations.

## The bases of human ocular development

### The early eye primordium is an outgrowth of the forebrain

In human embryos, the eye primordia develop as a subdomain of the prospective forebrain (prosencephalon), which can be distinguished morphologically by gestational day (GD)17 (reviewed in Creuzet and Etchevers 2017; Van Cruchten et al. 2017). Work in animal models has shown that the specification of eye fate is accomplished by a network of conserved transcription factors (TFs) that mobilize and coordinate core signaling pathways reiteratively used in organ morphogenesis. These genes are expressed in overlapping domains in the anterior neural primordium and their concerted activity specifies eye fate (reviewed in Beccari et al. 2013; Martinez-Morales 2016). Some of these will also be discussed in more detail further along in this review, as many are either regulated directly by Shh activity or affect its timing and effects during eye formation (Table 1).

**Table 1:** Human ocular developmental anomalies due to known mutations of genes functionally associated with SHH signaling

Disease	Ocular phenotype(s)	Gene(s) and/or Shh dosage-sensitive cell types	References
Aniridia	Aniridia, cataract with late-onset corneal dystrophy	*PAX6* – Optic vesicle	Jordan et al. (1992)
Axenfeld–Rieger syndrome	Myopia, microcornea, iris hypoplasia, corectopia, polycoria	*PITX2, FOXC1* – Neural crest cells	Semina et al. (1996); Nishimura et al. (1998)
Basal cell nevus (Gorlin–Goltz) syndrome	Myelinated fibers, optic nerve anomaly, epiretinal membrane, unilateral microphthalmia with cyst	*PTCH1, PTCH2, SUFU* – Optic vesicle and stalk	Johnson et al. (1996); Ragge et al. (2005b); Romano et al. (2011); reviewed in Onodera et al. (2017)
Congenital glaucoma and corneal dysgenesis	Congenital glaucoma	*CYP1B1, LTBP2, TEK, MYOC, SLC4A11, ADAM9, CPAMD8* *KERA, BCO2, TULP2* – Neural crest cells	Williams et al. (2017); Alsaif et al. (2018)
Curry–Jones syndrome	Iris coloboma, microphthalmia	*SMO* – Optic vesicle, neural crest cells	Twigg et al. (2016)
Donnai–Barrow syndrome	Large ocular globes (high myopia), coloboma	*LRP2* – Optic vesicle	Kantarci et al. (2007)
Grieg syndrome	Hypertelorism (one report of keratoconus)	*GLI3* – Neural crest cells	Wild et al. (1997); Démurger et al. (2015)
Holoprosencephaly	Anophthalmia, micro/anophthalmia, cyclopia, synophthalmia, microphthalmia, hypotelorism, coloboma	*SHH, PTCH1, CDON, DISP1, GAS1, GLI2, BOC*, (*LRP2*) – *BMP4, FGF8, FGFR1* – Optic vesicle	Belloni et al. (1996); Roessler et al. (1996); Wallis and Muenke (2000); Ming et al. (2002); Hayhurst and McConnell (2003); Schimmenti et al. (2003); Morcillo et al. (2006); Roessler et al. (2009); Bakrania et al. (2010); Ribeiro et al. (2010); (Rosenfeld et al. (2010); Gongal et al. (2011); Pineda-Alvarez et al. (2012); Hong et al. (2017); Richieri-Costa et al. (2016); Bruel et al. (2018) – Bakrania et al. (2008); Dubourg et al. (2016)
Ciliopathies Joubert syndrome – Oculocerebrorenal syndrome of Lowe – Micro/anophthalmia	Retinitis pigmentosa, retinal dystrophy, coloboma – Coloboma, congenital cataract – Coloboma, aphakia	*INPP5E, RPGRIPL1* ... – *OCRL* – *CCRK* – Optic vesicle	Bielas et al. (2009); Delous et al. (2007) – Zhang et al. (1995) – Lupu et al. (2018)
Opitz G/BBB syndrome	Hypertelorism, occasional coloboma	*MID1* – Neuroepithelium, neural crest cells	Halal et al. (1981); Quaderi et al. (1997); Pinson et al. (2004)
Smith–Lemli–Opitz syndrome	Cataract Blepharoptosis	*DHCR7* – Neural crest cells	Fitzky et al. (1998); Wassif et al. (1998)
Syndromic or isolated micro/anophthalmia	Micro/anophthalmia	*OTX2, SOX2, STRA6, VAX1, VSX2, RAX, PTCH1, ALDH1A3, RARB, GJA3*… also see *CCRK* above – Optic vesicle	Ragge et al. (2005a); Pasutto et al. (2007); Golzio et al. (2007); Chassaing et al. (2012); Slavotinek et al. (2012); Chassaing et al. (2016)

As the rostral prosencephalon bulges and initially splays at GD22, bilateral pits emerge on each side midway between the neural fold and floor plate, at an equivalent distance from the edge of the anterior neural fold (Cook et al. 2006; Creuzet and Etchevers 2017). These demarcate the future vesicles that will emerge toward the ectoderm over the next few days. By the time the optic vesicles have evaginated, they are already patterned along the proximal–distal (PD) axis, to give rise to the optic stalk proximally, and neural retina/retinal pigment epithelium (NR/RPE) distally.

By GD28, the optic vesicles have established full contact with the overlying ectoderm and a number of inductive events are triggered in each. This leads to the finer specification of the distal-most portion of the optic vesicle itself as the future neural retina, as well as the induction of lens fate in the overlying ectoderm. These inductive events are accompanied by extensive reshaping of the eye primordium, which then folds over itself to give rise to the optic cup, a bilayered structure in which the external layer will give rise to the RPE and the internal layer to the neural retina (reviewed by Martinez-Morales et al. 2017; Van Cruchten et al. 2017). In parallel to optic cup folding, the lens primordium invaginates and buds off the overlying ectoderm, to give rise to the lens vesicle. During this time, the entire forebrain primordium itself is growing in all dimensions. A flash animation of this dynamic process, to this point highly conserved among vertebrates and under control of homologous genes across species from fish to primates, can be found at the following link (Lamb et al. 2007): https://media.nature.com/full/nature-assets/nrn/journal/v8/n12/extref/nrn2283-s1.swf.

### Positional signals are exchanged within and between the ocular neuroepithelium and surrounding mesenchyme

An important population of mesenchymal cells intervenes between the prosencephalon and the overlying ectoderm, later separating the ectoderm from the thinning edge of the optic cup as it becomes the iris, and overlying the lens vesicle. It surrounds and is indispensable for the differentiation of the RPE and subsequently for the entire ocular globe and nerve as they form (Fuhrmann et al. 2000; Creuzet et al. 2005). This mesenchyme is mostly made up of cells known as the neural crest; in older literature it is called “mesectoderm”. Using fate-mapping techniques in model organisms such as chicken or fish embryos, neural crest cells (NCC) have been shown to delaminate from the dorsal neural folds of the caudal forebrain and rostral midbrain to surround the optic vesicles from both above and below to encase the prospective forebrain and eyes (Fuhrmann et al. 2000; Etchevers et al. 2001; Langenberg et al. 2008).

NCC initially outnumber the mesenchymal head mesoderm cells responsible for the formation of the first blood vessels on the dorsal aspect of the brain and eyes, but they rapidly co-mingle to provide a cellular matrix and organize into a primitive vascular network around the entire prosencephalic primordium and within the developing eye itself (Etchevers et al. 2001). NCC later infiltrate the iris to provide its pigmentation, and also differentiate into the corneal stroma and endothelium, the pericytes of the ocular choroid and hyaloid vasculature, as well as the viscoelastic sclera of the eyeball, orbital cartilage and bone, and oculomotor tendons (Johnston et al. 1979; Couly et al. 1993; Kastner et al. 1994; Zieske 2004; Gage et al. 2005; Grenier et al. 2009; Macé et al. 2011).

The NCC mesenchyme constitutes most of the structures of the face and neck around which the sense and secretory organs are organized, particularly the facial skeleton (Le Lièvre and Le Douarin 1975). Shh acts at multiple times on and during cephalic NCC differentiation into these and other structures as well as directly on the ocular primordia (Jeong et al. 2004; Lan and Jiang 2009; Aoto and Trainor 2015). Both posterior cephalic NCC and Shh activity are critical for cardiac outflow tract morphogenesis during heart development (Kirby et al. 1983; Dyer and Kirby 2009). These observations explain clinical associations of facial dysmorphic traits such as hypotelorism, agnathia or cleft lip/palate, or cardiac defects, with ocular developmental anomalies (ODA), some of which had been described many decades before the molecular bases were established (DeMyer et al. 1964; Jeong et al. 2004, 2006; Pasutto et al. 2007; Golzio et al. 2007; Chassaing et al. 2009; Aoto and Trainor 2015).

By the beginning of gestational week 8 (GW8), the optic stalk and the optic cup are fully formed, and the differentiation of the lens as well as the retina is well underway (Van Cruchten et al. 2017). These processes are controlled both directly and indirectly by Shh in the different primordia. Notably, periocular NCC interact with the forming cornea and lens by modulating and transducing Wnt and Tgfb signaling pathways as well as in defining the edge of the optic cup from which the ciliary body, Schlemm’s canal and iris will form (Ittner et al. 2005; Aguiar et al. 2014).

The morphogenetic events leading to optic cup folding also induce the formation of a perpendicular fold, running along the ventral proximo-distal axis of the optic primordium. This opening, called the optic or choroid fissure, runs along the future optic stalk and also comprises the ventral retina. A transient structure as such, it is the conduit to establish vascular irrigation of the differentiating organ, and subsequently guides the exit of retinal ganglion cell axons to innervate the visual centers in the developing brain as the future optic nerve forms (Patel and Sowden 2017). At its most distal aspect, the fissure also attains the iris. Eventually, it will fuse all along its length to generate the fully mature optic nerve, retina and anterior chamber. Defective choroid fissure closure leads to various degrees of coloboma. Again, Shh seems to play a determinant role in this process (Morcillo et al. 2006; Gongal et al. 2011). In humans, the fissure closes around the seventh week of gestation (Barishak 1992).

Further differentiation of RPE cells give rise to a squamous, pigmented epithelium, while differentiation of the neural retina gives rise to the laminated circuit of neurons involved in light reception and processing (reviewed in Creuzet and Etchevers 2017; Van Cruchten et al. 2017). RPE function is critical for the survival and appropriate connectivity of the overlying retinal photoreceptor layer and remains so throughout life—RPE malfunction, as can happen in retinitis pigmentosa, leads to retinal dystrophy (McKechnie et al. 1985; Zhao et al. 2011). These differentiation events continue throughout gestation and throughout the first year after birth, and Hh pathway signaling remains important at multiple points in these processes.

### Any given ODA has multiple specific causes

Interrupting any of the steps in ocular development can lead to a range of overlapping pathological outcomes. Brain malformations affecting the future telencephalon and diencephalon are often associated with severe ODA—primary anophthalmia is found with anencephaly and thereby incompatible with postnatal life; severe microphthalmia resulting in secondary anophthalmia (micro/anophthalmia), nanophthalmia and cystic eye are found in syndromes also affecting the structure of the anterior brain (prosencephalon) which will give rise to the cerebral hemispheres of the telencephalon and the hypothalamic–pituitary axis of the diencephalon, and can thus be found associated with combined pituitary hormone deficiencies. These are regularly found with mutations of *OTX2*, an evolutionarily conserved TF gene highly important for anterior head development (Acampora et al. 1995; reviewed in; Slavotinek 2011). Phenotypic outcomes, even within the same family, of *OTX2* mutations range from subclinical to micro/anophthalmia but also include otocephaly and dysgnathia (Ragge et al. 2005a; Chassaing et al. 2012). *Otx2* is an active Shh target that is expressed in the ventral optic primordium and subsequently becomes a master regulator of RPE differentiation, necessary for survival and persistence of the optic cup (Martínez-Morales et al. 2003, 2004; Hever et al. 2006; Halilagic et al. 2007; Larsen et al. 2009; Westenskow et al. 2009).

Aphakia results from the interruption of any signals involved in optic cup invagination, lens induction, differentiation or survival—if early enough, the absence of lens will have repercussions for the remaining optic vesicle. Congenital cataract results from affecting later lens differentiation and secondary fiber formation and matrix composition. NCC are indispensable for anterior chamber development, particularly the cornea, iris, trabecular network and ciliary process (reviewed by Williams and Bohnsack 2015; Fuhrmann et al. 2000), associated with myopia, microcornea, iris hypoplasia, corectopia, polycoria and other signs of Axenfeld–Rieger syndrome or congenital glaucoma. NCC are also necessary for the differentiation of the choroid, and they constitute the sclera and attachment tendons as well as contribute to the innervation of the extraocular muscles, by which they may play a part in congenital nystagmus or strabismus (Creuzet et al. 2005; Grenier et al. 2009; reviewed in; Williams and Bohnsack 2015). However, when intervening between prospective lens and the distal point of contact with the ectoderm, ectopic NCC are associated with inhibition of lens formation and differentiation (Sullivan et al. 2004).

Correct optic stalk elongation and induction of the RPE are associated with the differentiation of the lens. In chick embryos where the Pax6 TF was inhibited experimentally, severe colobomas are found with remnants of ectopic RPE, in the absence of lens or an appropriately laminated neural retina (Canto-Soler and Adler 2006). Malformations of the resultant optic nerve, either due to coloboma or problems in retinal ganglion cell axonal growth to the brain, are visible in human pathology as leukocoria.

## Hedgehog signaling in early brain and ocular pathogenesis

### The holoprosencephaly spectrum impacts eye formation and is the result of SHH signaling deficiency

The Hh signaling pathway is essential for many of the steps leading to the formation of a mature ocular primordium, described above. SHH itself is one of three proteins of the hedgehog protein family in humans (including Sonic, Desert and Indian hedgehog; Pandit and Ogden 2017), and has been the most studied as it appears to play the most varied roles in organogenesis around the body. In humans, *SHH* is located on chromosome 7q36.3 and the only relevant ligand from this family during embryonic forebrain development (Odent et al. 1999). During these early stages, the role of SHH in controlling eye patterning is intimately linked to its function directing the specification of ventral brain structures. Shh is expressed along the axial mesoderm (the prechordal plate and notochord) and the neural floorplate where it is particularly essential to pattern ventral neural tissues (Krauss et al. 1993; Ekker et al. 1995a; Chiang et al. 1996; Schauerte et al. 1998; Odent et al. 1999). Later, human SHH is transcribed in the superficial lens and posterior retina during neural differentiation while it continues to be produced in the telencephalic floorplate (Bakrania et al. 2008).

As demonstrated in mice, under the influence of midline Hh activity, transcription of the evolutionarily conserved TF *Pax6* normally becomes downregulated in the medial eye field, and these cells in turn transcribe *Pax2* (Chiang et al. 1996). As a consequence, two lateral domains of *Pax6* expression are generated. The epithelia proliferate and subsequently evaginate from the lateral walls of the forebrain to give rise to the optic pits and eventually the optic vesicles. Loss or downregulation of Hh activity interferes with these early stages of eye as well as forebrain patterning and has been associated both in humans and animal models with a defect called holoprosencephaly (HPE). This malformation has been classically defined as the failure of the forebrain to divide into two cerebral hemispheres. Clinically, this covers a phenotypic spectrum in the brain that always features ocular and related craniofacial malformations, which can range from complete anophthalmia to cyclopia to synophthalmia to microphthalmia, hypotelorism and coloboma (Wallis and Muenke 2000; Hayhurst and McConnell 2003; Gongal et al. 2011). Loss-of-function mutations in *SHH* can occasionally be associated with isolated coloboma along the spectrum. In one family recently described, a proband presented with unilateral iris coloboma and microcephaly but no HPE, the mother had bilateral iris coloboma, while her brother had multiple malformations compatible with unconfirmed HPE (Bruel et al. 2018).

HPE is relatively common in early embryonic life, but not always compatible with birth, by which time HPE manifests as a rare and sometimes underdiagnosed condition as a result of this phenotypic as well as genetic heterogeneity (Dubourg et al. 2016).

### The complexity of Hh signaling transduction is reflected in a continuous range of pathological phenotypes

After the discovery that HPE results from loss-of-function mutations in *SHH* (Belloni et al. 1996; Roessler et al. 1996), subsequent research showed that HPE is genetically very heterogeneous. The direct pathway effector genes *PTCH1* (Ming et al. 2002; Ribeiro et al. 2006), *CDON* (Bae et al. 2011), *DISP* (Roessler et al. 2009), *GAS1* (Ribeiro et al. 2010; Pineda-Alvarez et al. 2012), and *GLI2* (Roessler et al. 2003), as well as other genes also cause various forms of HPE either alone or in compound heterozygous combination (Dubourg et al. 2016; Mouden et al. 2016). Human mutations in *LRP2*, a non-specific co-receptor of SHH also known as megalin, can lead to Donnai–Barrow syndrome, in which large ocular globes can be associated with high myopia and partial colobomas, in contrast to *Lrp2*-mutant mice presenting micro/anophthalmia (Kantarci et al. 2007). One study found that two HPE microform cases with large genomic deletions included the *LRP2* gene among others (Rosenfeld et al. 2010).

Newer identified components of the HH signaling pathway, such as *BOC*, seem to be phenotypic modifiers of HPE caused by *SHH* mutation. Indeed, *Boc* mutants in mouse do not display HPE, but the allele exacerbates HPE phenotypes when combined with mutations in *Cdon* or *Gas1* (Zhang et al. 2011; Seppala et al. 2014). Moreover, missense *BOC* variants have recently been found in HPE patients, supporting a potential modifier function for *BOC* in human HPE (Hong et al. 2017).

**Fig. 1:**
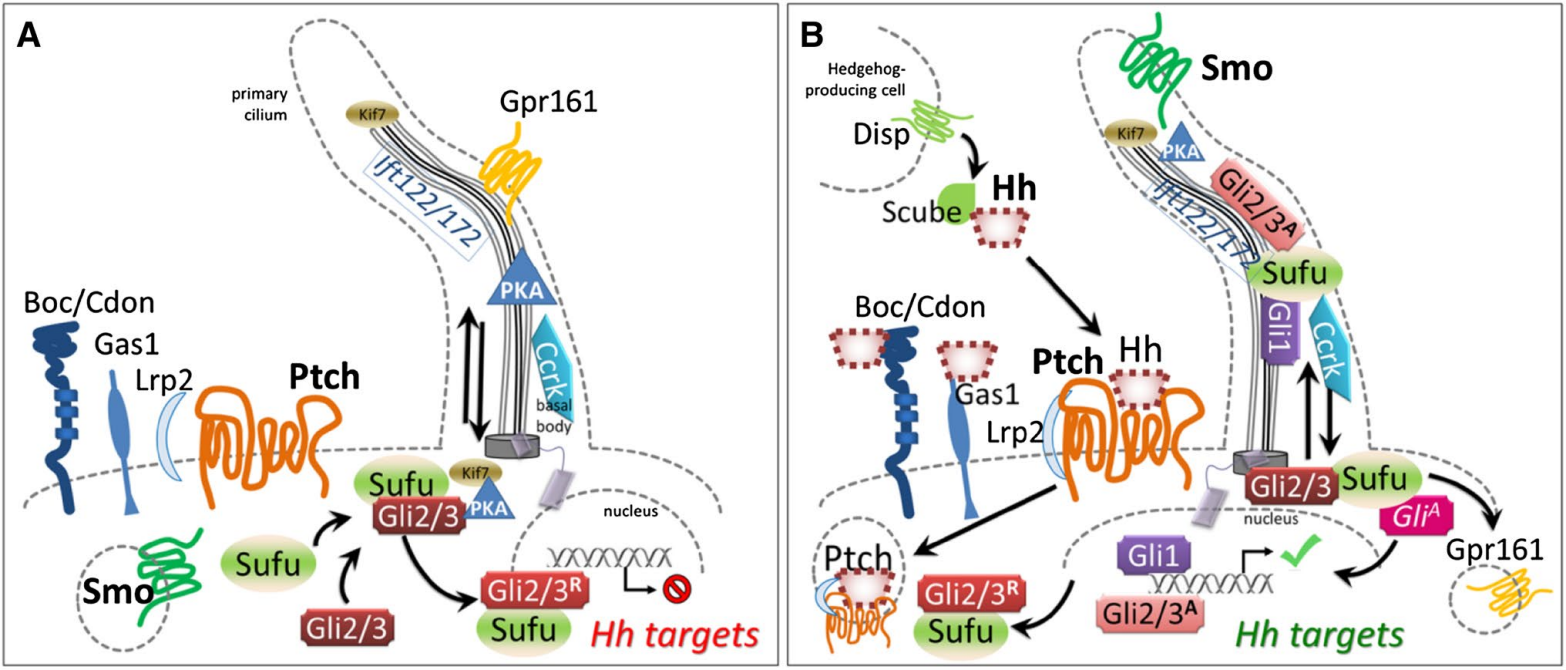
**a** Before binding by a Hedgehog family ligand (most often Shh, but also Ihh or Dhh), Ptch1 and presumably Ptch2 maintain Smo sequestered, by a currently unknown mechanism. Under these circumstances, both anterograde and retrograde transport in functionally intact primary cilia is necessary to allow PKA-dependent cleavage of the Gli3 transcription factor to its repressor form and its shuttling from the base of the cilium to target DNA, accompanied by Sufu. The same mechanism may govern Gli2 processing. **b** Hedgehog proteins are post-translationally modified; the secretion and multimeric conformation of Shh is organized by the Disp transmembrane receptor in signaling cells, accompanied by Scube. Binding of Hh to Ptch receptors leads to internalization and degradation, and relocalization of Smo to the primary ciliary membrane, which changes lipid composition. Other receptors include Boc, Cdon and Gas1, which may refine signal integration. Lrp1 is a non-specific but necessary co-receptor. Hh binding leads to ciliary clearance of the G-protein coupled negative regulatory receptor Gpr161, reduction in PKA production, and accompaniment by Sufu of immature Gli2/3 proteins along the ciliary microtubules for their requisite processing to activating forms. Gli1 is transcribed as a Shh target and positively reinforces this cycle, while Ptch1 itself is another target whose increased transcription sharpens thresholds and raises cellular stakes for survival, as continued Hh stimulation becomes necessary for the target cell. The activity of cell cycle-related kinase (Ccrk) is necessary for ciliary integrity and acts between Smo and Gli2 in effecting readout of Shh levels within the optic primordium

Ultimately, mutations in these HPE-causing genes, with their variable effects on the early eye primordium, seem to globally dampen Shh signaling within the forebrain and ocular anlage. However, this overview may be too simplistic (Fig. 1). In addition to a balance of effector transcription factors of the Gli family, with both repressor and activator activities in specific context, there are multiple points at which the Shh pathway interacts, both as target and as modulator, with branches of other signaling pathways. These include bone morphogenetic protein (BMP), fibroblast growth factor (FGF) (Ohkubo et al. 2002) and the vitamin A metabolite, retinoic acid, where signaling to transcriptional targets affect the fates of the same tissues and cause overlapping syndromic malformation phenotypes. Indeed, *BMP4* (Bakrania et al. 2008), *FGF8* and its receptor *FGFR1* (Dubourg et al. 2016), and *STRA6*, an obligate mediator of RA signal transduction (Pasutto et al. 2007; Golzio et al. 2007; Kawaguchi et al. 2007), have all been identified as responsible for syndromes comprising forebrain malformation and severe micro/anophthalmia. As for Shh itself, each phenotypic spectrum begins at subclinical. These pathways are presented in more depth in other reviews of this issue.

### Transcriptional targets of Shh signaling mediate ocular morphogenesis: early stages

An important consequence of Hh signaling is the direct or indirect control of a number of TFs that establish positional identity within the ocular primordium. Comprehensive presentation of all potential effectors is beyond the scope of this review, but some important ones are mentioned here. At early stages, midline Hh influences the establishment and maintenance of identity along the proximo-distal axis, which will later translate into the difference between the ocular globe itself and the nerve and chiasm.

Transcriptional repression of the *Pax6* TF and concomitant induction of *Pax2* in the medial portion of the eye field by early Hh activity at the ventral midline is not only required for the splitting of the eye field, but it also has an essential role in patterning the evaginating optic vesicle along the proximal–distal (PD) and dorsoventral axis in all vertebrates studied. As the optic vesicles grow, they keep this complementary expression, so that the most proximal region keeps expressing *Pax2*, while the distal-most region expresses *Pax6* (Macdonald et al. 1995; Schwarz et al. 2000). The Pax2+ region of the primordium remains always in direct contact with the embryonic ventral midline and maintained by Shh emanating from this region (Ekker et al. 1995b; Macdonald et al. 1995). Pax2 and Pax6, in turn, engage in a cross-repressive interaction that maintains the boundary between proximal and distal domains in the optic primordium (Schwarz et al. 2000). A recent paper shows that Shh also induces the expression of *midline-1* (*mid1*) in the optic stalk. *mid1* encodes a E3 ligase involved in the ubiquitination and proteasomal degradation of Pax6 (Pfirrmann et al. 2016). In this way, higher levels of Shh activity in the optic stalk ensure this region is depleted of residual Pax6 protein in a timely fashion. Mutations in human *MID1*, normally expressed throughout the early developing brain, lead to Opitz G/BBB syndrome, which is characterized by hypertelorism, oesophagolaryngotracheal defects and hypospadias and is also occasionally associated with coloboma (Halal et al. 1981; Pinson et al. 2004).

Shh is chemotactic for chick craniofacial NCC in vitro; an increasing gradient may guide NCC towards the optic stalk as well as toward the ventral prechordal plate, structures each expressing Shh at appropriate stages (Tolosa et al. 2016). This periocular mesenchyme later induces the development of the RPE and anterior chamber, further repressing expression of Pax6 and other neural retina-specific TFs in the intermediate area (Fuhrmann et al. 2000).

### Gradients of Shh confer positional coordinates on cells of future retina

At a slightly earlier stage, while the proximo-distal (PD) axis of the evaginating optic vesicle is determined by midline Shh signaling, the primordium also gets patterned along the nasal/temporal (NT) and dorsal/ventral (DV) axes. Zebrafish optic vesicle evagination occurs in a very reduced window of time as compared to this process in mammals. The eye field becomes split in two domains by the same morphogenetic movements driving the lateral evagination of the optic vesicles (Rembold et al. 2006; Ivanovitch et al. 2013). Determination of PD and NT domains occurs simultaneously (Picker and Brand 2005; Picker et al. 2009). As the optic vesicle matures into an optic cup, the ventral connection of the optic vesicle with the neural tube narrows down to a small region located at the most anterior part of the eye primordium to give rise to the optic stalk, and the whole vesicle rotates anteriorly to acquire its final position, in which the NT axis is aligned with the embryonic anterior-posterior (AP) axis (Li et al. 2000). Recent studies in zebrafish have shown that the establishment of nasal and temporal coordinates occurs at the very onset of optic vesicle evagination, as early as or earlier than determination of the PD axis, under the influence of Shh (Hernandez-Bejarano et al. 2015).

Thus, at early stages in zebrafish embryos, the prospective temporal region of the optic vesicle is both continuous with the ventral midline and directly under the influence of Shh. Shh drives the expression of the temporal fate determinant TF *foxd1* in this region (Hernandez-Bejarano et al. 2015). In mammals, morphogenesis of the optic cup may not involve as complex spatial rearrangements, since the NT axis seems to be specified already aligned with the AP axis of the developing embryo, in the models commonly studied. Nonetheless, it is likely that the Hh signaling pathway has a similarly determinant role in the formation of the temporal region of the mammalian eye primordium. The temporal region of the eye is specified next to prospective hypothalamic territory, a source of SHH in humans as well as other vertebrates (Odent et al. 1999), and the expression of ocular *Foxd1* is first seen at optic vesicle stages in the posterior, future temporal half of the eye primordium in mouse embryos (Hatini et al. 1994; Carreres et al. 2011). Reciprocally, Foxd1 may in turn maintain *Shh* expression, as suggested by the phenotype in *foxd1* mouse mutants, in which ventral *Shh* is lost, associated with ventral ocular defects (Huh et al. 1999).

PD patterning in vertebrates is also directly linked to DV fate. Like Pax2, the Vax1 and Vax2 TFs are known downstream targets of Shh expressed specifically in the optic stalk (Hallonet et al. 1999; Barbieri et al. 1999; Bertuzzi et al. 1999; Ohsaki et al. 1999; Take-uchi et al. 2003; Lupo et al. 2005; Zhao et al. 2010). Not only is optic nerve differentiation arrested by Shh insufficiency (Macdonald et al. 1997; Bertuzzi et al. 1999; Barbieri et al. 2002; Take-uchi et al. 2003), but ventral optic cup specification and optic fissure fate as well (Hallonet et al. 1999). Ultimately, consistent with its role as a SHH signaling mediator and similar to the mouse mutant phenotype, a human *VAX1* mutation has been found in a patient with microphthalmia in association with cleft lip/palate (Slavotinek et al. 2012). *VAX2* haploinsufficiency may also be associated with subtler ocular defects related to PD patterning and subsequent retinal dystrophy (Norgett et al. 2015).

In summary, coordination of PD, DV and NT identities is indispensable to later optic vesicle persistence and organization of the various cell types differentiating therein. Defects in any of the patterning processes during the first two months of gestation in humans result in misshaped primordia, leading to microphthalmic and coloboma phenotypes.

### Patched (PTCH) receptors are key gatekeepers of Shh signal transduction

The transmembrane protein PTCH1 is both a receptor for SHH and a highly conserved transcriptional target and readout of the hedgehog pathway, from insects to mammals (Goodrich et al. 1996). This receptor works in an unusual way, since SHH binding results in its degradation, and relieves its constitutive repression of Smo, which is the critical transmembrane transducer of SHH signal into the cytoplasm (Fig. 1). Indeed, absence of Patched activity results in widespread transcriptional activation of other targets of Shh signaling.

Ptch receptors are known as “dependence receptors”—when bound, they allow cell survival but once having transduced signal, in the absence of hedgehog proteins, they cause apoptosis (Thibert et al. 2003; Fombonne et al. 2012). In humans, there are two homologues: *PTCH1* on 9q22.32, and *PTCH2* on 1p34.1. Unbound PTCH1 inhibits signaling through SMO by blocking its associations with intracellular proteins and subsequent phosphorylation. SMO inhibition can also be mimicked by the use of the direct SMO antagonist, cyclopamine (Chen et al. 2002). Both human and mouse cells receptive of Shh signaling, once exposed to Shh, then become dependent on it for their survival. Subsequent lack of Shh induces caspase-9 ubiquitination and subsequent programmed cell death (Fombonne et al. 2012).

By this mechanism, Shh is particularly vital for the maintenance of multipotent periocular and craniofacial NCC viability (Ahlgren and Bronner-Fraser 1999; Jeong et al. 2004; Calloni et al. 2007). In the upper jaw, epithelial Shh signals through Smo to the mesenchyme, maintaining cyclin D2 and palate cell proliferation during shelf outgrowth (Lan and Jiang 2009). This sensitivity of facial NCC to Shh availability underlies at least in part, the frequent association of cleft palate and ODA and/or HPE. The NCC mesenchyme of the first pharyngeal arch in all vertebrate embryos gives rise to both a rostral/dorsal maxillary component, precursor to the palate, and a caudal/ventral mandibular component, precursor to the lower jaw. Indirectly, SHH may also mediate the local effects of *OTX2* mutations on both eye and jaw in human micro/anophthalmia with or without otocephaly and micro/agnathia (Chassaing et al. 2012).

**Fig. 2:**
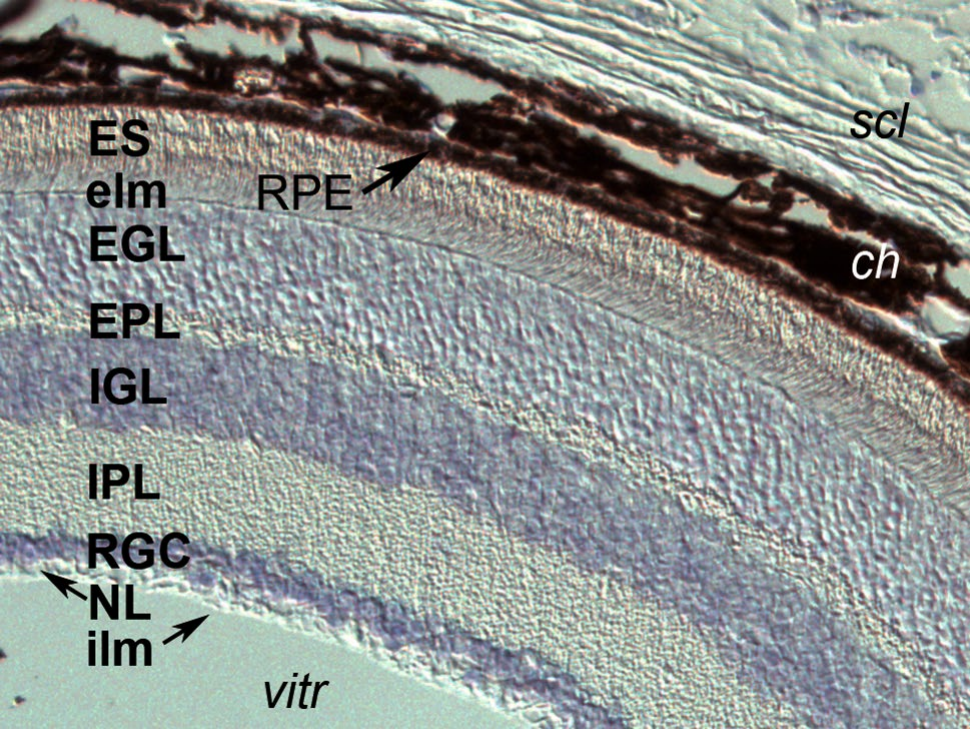
*Ptch1* mRNA expression in the adult mouse retina (as reported in Chassaing et al. 2016). Transcripts in bluish-purple are present within all cell bodies of the retina. From external to internal:* scl* sclera,* ch* choroid plexus, heavily invested with melanocytes,* RPE* retinal pigmented epithelium,* ES* external segment of photoreceptors—both rods and cones in contact with the RPE,* elm* the external limiting “membrane” (aligned tight junctions of photoreceptors and Müller cells),* EGL* external granular layer, composed of photoreceptor soma,* EPL* external plexiform layer, which contains the dendrites of bipolar and horizontal interneurons that integrate photoreceptor signals,* IGL* internal granular layer, with the cell bodies of the bipolar, horizontal and amacrine cells,* IPL* internal plexiform layer, with the axons of bipolar cells connected to amacrine cells and retinal ganglion cell (RGC) dendrites,* RGC* cell body layer,* NL* nerve fiber layer of RGC axons converging on the optic nerve,* ILM* inner limiting membrane,* vitr* vitreous

Both homozygous knockout and overexpression of *Ptch* in mice lead to embryonic lethality (Goodrich et al. 1996). *PTCH1* mutations and deletions have been identified in patients with a dominant form of syndromic and variable expression of HPE, comprising infrequent cleft lip/palate (Ming et al. 2002; Richieri-Costa et al. 2016). Other inactivating variants may contribute to nonsyndromic CLP (Cobourne et al. 2009). However, relative to ocular development, mutations in *PTCH1* have been identified to be a major cause of human micro/anophthalmia or anterior segment dysgenesis (Chassaing et al. 2016; Richieri-Costa et al. 2016). *Ptch1* continues to be expressed in the adult mouse retina (Fig. 2). Families with one type of ocular malformation can present individuals with the other and/or both, reflecting great clinical heterogeneity within this class of ocular developmental anomalies as well as relative to other craniofacial effects of such mutations.

To make the clinical picture even more complex, somatic loss-of-function mutations in either *PTCH1* or *PTCH2* are among the multiple genetic causes of basal cell nevus (BCN) syndrome (Bresler et al. 2016). The result of such mutations is overactivation of Shh pathway transduction. Homozygous loss-of-function mouse embryos show dramatic ventralization of the CNS, with total repression of *Pax6* expression and absent optic evaginations (Zhang et al. 2017). Ectopic and sustained Shh expression in mouse facial epithelia leads to cleft lip/palate, hypodontia and hypertelorism (Cobourne et al. 2009). *PTCH1*/*2* mutations that have been identified in both BCN syndrome and HPE are usually missense variants and dominant, implying that additional haploinsufficiency also would be embryonic lethal in humans. A truncating, dominant germline mutation in *PTCH1* was identified in one BCN syndrome patient with myelinated fibers and optic nerve anomalies in one eye and epiretinal membranes in both (Romano et al. 2011). Another patient with a truncating *PTCH1* mutation was reported with both BCN syndrome and unilateral microphthalmia with cyst (Ragge et al. 2005b). For the moment, no *PTCH2* mutations have been associated with ODA.

These varied effects are likely the result of the complex coordination of eye morphogenesis and the differentiation of its critical but more superficial tissues. Non-autonomous cell–cell communication between the epithelial (retinal, lens) and mesenchymal (neural crest) eye progenitors is essential for the correct induction and patterning of each, as has been examined in numerous animal models over the years. Such studies have been a source for candidate genes and pathways in human ODA pathogenesis.

Indeed, ODA resulting from interference with growth and differentiation of the optic vesicle, stalk and disc are genetically heterogeneous, and the major causative genes, *PTCH1* and *SOX2*, account together for at most one in five micro/anophthalmia cases (Chassaing et al. 2016). *SOX2* is a positive upstream regulator of SHH signaling (Zhao et al. 2012). Interestingly, nearly all the other genes that had been identified to date in severe ODA and anterior segment dysgenesis are also transcription factors, often found to work in gene regulatory networks. Another syndromic ODA gene, *STRA6*, is an apparent exception to this observation (Pasutto et al. 2007; Golzio et al. 2007). *STRA6* encodes a ubiquitous membrane protein enabling the intracellular passage of vitamin A, the metabolic precursor to retinoic acid (RA) (Kawaguchi et al. 2007). RA, like SHH, is another extremely important developmental signaling molecule but, unlike SHH, acts directly in concert with nuclear receptors to control transcriptional regulation, including that of *SHH* itself (Rhinn and Dollé 2012). Teratogenic or experimentally induced changes in RA levels at early stages of embryogenesis have dramatic effects for the establishment of the AP axis of the whole organism. In the context of the murine eye, prevention of RA signaling in NCC alone, by conditional knockout of all three heterodimeric nuclear receptors necessary for its transduction, leads to frontonasal truncation, eyelid agenesis and severe microphthalmia, with loss of the ventral retina and optic nerve (Matt et al. 2008; Dupé and Pellerin 2009). Double-germline mutants in RA receptor subunits also show malformations of the anterior chamber, aphakia, congenital cataract and retinal colobomas, attributed to ectopic NCC (Mark et al. 1998). The effects of RA deficiency in the eye may be partly mediated by disrupting the integration of Shh signaling at the transcriptional level, perhaps potentiating it.

## Hedgehog signaling in malformations comprising later ocular derivatives

### SHH genotype–phenotype correlations in ocular developmental anomalies

At later stages of optic cup maturation, Shh is required for RPE differentiation (Zhang and Yang 2001; Perron et al. 2003; Dakubo et al. 2008), choroid fissure formation and closure, and optic nerve maturation (Dakubo et al. 2003; Morcillo et al. 2006). Continued Shh signaling from the hypothalamus is necessary for optic disc maturation, the site through which retinal ganglion cell axons project into the optic nerve. Reduced signaling from this specific source, as engineered by conditionally driving *Shh* inactivation with a Cre recombinase under control of a hypothalamic-specific Shh regulatory element, leads to hypoplastic optic nerve in mouse models (Zhao et al. 2012).

Most mutations in *SHH* associated with HPE cases are not in the C-terminal region. However, these are also phenotypically heterogeneous or show incomplete penetrance within families. HPE-causative mutations in *SHH*, including in the C-terminal region, as well as mutations in other pathway effectors can also lead to more subtle defects not always diagnosed in addition to uveal and chorioretinal coloboma and microphthalmia, including refraction errors induced by myopia, retinal thinning, malposition of the optic nerve, and borderline microcornea (Pineda-Alvarez et al. 2011).

Occasionally, mutations in *SHH* have been reported in milder, isolated ODA cases presenting microphthalmia and/or coloboma (Schimmenti et al. 2003; Bakrania et al. 2010). During post-translational processing, the hedgehog protein precursor undergoes autocatalytic internal cleavage, yielding an active N-terminal domain covalently attached to cholesterol and palmitate, and a C-terminal domain necessary for that attachment (Porter et al. 1996). The mutations found in these isolated ODA patients lead to the deletion of eight amino acids in the C-terminal autocatalytic domain of SHH and may interfere with lipid attachment. The effect is incompletely penetrant: of family members in both these reports who were carrying such mutations, some were entirely affected, others had unilateral or partial colobomas, while one proband had bilateral microphthalmia in addition to severe uveoretinal coloboma. Defective lipid attachment is likely to have dramatic consequences for SHH signaling. Cholesterol annexation is essential for a fully functional Shh molecule and establishing tissue-appropriate titers (e.g., Huang et al. 2007; Dessaud et al. 2008). Recent reports suggest that cholesterol may be able to also modulate the activity of the pathway by directly binding to a hydrophobic groove in the extracellular portion of Smo (Luchetti et al. 2016; Byrne et al. 2018). Smith–Lemli–Opitz syndrome (SLOS), in which cataracts and blepharoptosis are frequent among a wide constellation of congenital anomalies attributed to reduced SHH signaling, is caused by mutations in *DHCR7*, which encodes a rate-limiting enzyme in the cholesterol biosynthesis pathway (Wassif et al. 1998; Fitzky et al. 1998). The subsequent cholesterol deficit in SLOS impairs Smo translocation within the lipid membrane to the primary cilium (Blassberg et al. 2016), as discussed further below.

It seems likely that pathway effector mutations in humans will increasingly be found to be pathogenic concerning later outcomes of maturation of specific tissue components of the eye. Indeed, many ciliopathies discussed briefly below, may be imputed in part to such roles for Shh signaling.

### DHH and IHH may affect later corneal and retinal maintenance

Other Hedgehog family members in humans include Desert hedgehog (*DHH*, on 12q13.12) and Indian hedgehog (*IHH*, on 2q35). Although Dhh has been reported to be the major ligand present and involved with corneal homeostasis in the adult mouse, it has not to date been implicated in human ODA or later ocular phenotypes (Kucerova et al. 2012). Mouse null mutants in *Ihh* have no reported ocular defects, although they die shortly after birth with numerous skeletal anomalies, mildly foreshortened snout and mandible and a rounded skull (St-Jacques et al. 1999). Pathogenic *IHH* mutations in humans, consistent with these important roles demonstrated in endochondral ossification, tend to be missense mutations causing acrocapitofemoral dysplasia or brachydactyly type A1, but do not affect the eye (Byrnes et al. 2009). However, in addition to *Shh*, both *Dhh* and *Ihh* are expressed in the postnatal rat retina. Ihh secretion by choroidal endothelial cells impacts both the formation of the RPE inside, and the sclera outside, of this meningeal-like layer (Dakubo et al. 2008). We predict that future functional as well as malformative roles of other hedgehog signaling ligands will be discovered in human ocular pathology.

### *DISP1* mutations indirectly affect the eyes

*DISP1* (dispatched Resistance Nodulation Division transporter family member 1), in contrast to most other molecules reviewed here that are present in target tissues, encodes a protein needed for the secretion of the mature SHH peptide (Tian et al. 2008; Tukachinsky et al. 2012, Fig. 1). Mouse *Disp* mutations lead to HPE and misplaced early eye primordia (Tian et al. 2008). However, human mutations in *DISP1* are rarely diagnosed in HPE and have been reported in microforms with incomplete penetrance, at the level of the eyes showing hypotelorism and upslanting palpebral fissures (Roessler et al. 2009); other patients with compound heterozygous *DISP1* mutations sometimes in combination with other HPE gene mutations, tend to only have hypotelorism in conjunction with lobar or microform HPE (Dubourg et al. 2016; Dupé et al. 2017).

It is interesting that patients carrying deletions of the 1q41q42 region including *DISP1*, have rather opposite symptoms, again affecting tissues around the eyes—hypertelorism, deep-set eyes, some effects on the palpebral fissures, and frontal bossing, but no clinical HPE (Shaffer et al. 2007). Such signs, evocative of a subset of BCN syndrome symptoms, are the Shh hyperactivation counterpart to the cranial midline hypoplasias seen in HPEs. They may reflect the fact that genomic regulatory elements are lost as well as *DISP1* haploinsufficiency or paradoxical effects on the repressor/activator balance of effector transcription factors of the GLI family. Ultimately, although implicated in human ocular pathology, *DISP1* mutations and truncations affect SHH signal transduction in an incomplete manner and do not phenocopy even partial *SHH* loss-of-function.

### GLI proteins are context-dependent transcriptional activators and repressors

The three members of the GLI family of proteins (glioma-associated oncogene family zinc finger; homologous to the single *Drosophila* segment polarity protein Ci, for “cubitus interruptus”) are the direct transcriptional effectors of Shh signaling. The three homologues in humans, *GLI1, GLI2* and *GLI3*, are on chromosomes 12q13.3, 2q14.2, and 7p14.1, respectively. These TFs act in both repressor and activator forms, post-transcriptionally dependent on Hh signaling activity. This dual role was first established in *Drosophila* (Méthot and Basler 1999). As an activator (in response to Hh), Ci directly controls *ptc* transcription, and this role is evolutionarily conserved through to mammals—*Ptch1* is often used as a readout of Shh activity (Goodrich et al. 1996).

*GLI3* has been particularly well-studied. In mice, *Gli3* is normally expressed in the optic stalk. *Gli3* knockout mutants develop microphthalmia, with reduction in the *Pax6*-expressing distal domain of the optic vesicle, and concomitant upregulation of *Pax2* and *Vax1*/*2* TFs in the stalk, which thickens. *Otx2* expression in the ventral optic cup is also reduced secondarily (Aoto et al. 2002). Interestingly, these effects phenocopy the effects of augmenting Shh signaling in the optic primordium and translate the loss of the Gli3 repressor form in particular (Gli3-R).

However, the activator form of GLI3 is also necessary for human health, though not implicated specifically in eye formation. Pallister–Hall syndrome (PHS) is a rare, often lethal condition comprising massive benign hamartomic tumors of the hypothalamus, effects on the pituitary gland and subsequent adrenal function, and effects on digit numbers (polydactyly or syndactyly). Such effects on digits had been anticipated in experimental embryology by directly or indirectly manipulating Shh levels in the outgrowing limb (Niswander et al. 1994), many years before the other signal transducers in the cascade had been identified, much less in human pathology. The dominant truncating mutations in *GLI3* that cause PHS result in constitutive availability of the GLI3-R form, and a consequent imbalance relative to its activator form (Démurger et al. 2015, Fig. 1). However, other mutations, including large deletions, leading to *GLI3* haploinsufficiency are associated with the distinct Grieg syndrome, also including poly/syndactyly, but characterized by different craniofacial abnormalities, such as macrocephaly (occasionally associated with macrosomia) and hypertelorism. Further abrogation of *GLI3* may be embryonic lethal even earlier in humans, such that its potential ocular phenotype would not have been reported.

A major gene of Axenfeld–Rieger syndrome responsible for anterior segment dysgenesis and congenital glaucoma when mutated, the *FOXC1* transcription factor (Nishimura et al. 1998), is a known SHH signaling target in the periocular mesenchyme. Recently, it has been found that FOXC1 directly activates the transcription of GLI2-mediated SHH targets in human basal-like breast cancer cells by binding and acting as a co-factor to GLI2, bypassing SMO (Han et al. 2015). This is but one example that the contextual interplay between the many effector TFs directly and indirectly integrating SHH signaling effects on eye morphogenesis merits further work.

### Mutations in *SMO*, the critical effector of Shh signal transduction, can have repercussions for the anterior chamber

Smoothened (Smo) is a G protein-coupled receptor that partners intracellularly with scaffolding proteins such as Suppressor of Fused (Sufu, discussed below) to relieve the constitutive processing of Gli TFs into their N-terminal truncated repressor forms, and allow their transcriptional activator forms to predominate. Cyclopamine is a historic and well-known Hh signaling antagonist acting on SMO, which is initially bound and repressed by PTCH1 at the cell membrane (Taipale et al. 2000). The identification of cyclopamine as the teratogenic mediator of cyclopia and severe alobar HPE in pregnant animals grazing on *Veratrum californicum* (false hellebore or corn lily) dates to the mid-twentieth century (James 1999).

A few patients with presumably heterozygous deletions comprising the *SMO* locus at 7q32.3 have been reported. These mutations did not lead to even minor forms of HPE, but their clinical spectrum was not further delineated (Rosenfeld et al. 2010). Of the nine patients reported with heterozygous deletions including the *SMO* locus in the DECIPHER database (https://decipher.sanger.ac.uk), seven have no ocular symptoms aside from strabismus, while patient 258,033 with a nearly 7-Mb deletion comprising 74 genes has an epibulbar dermoid tumor, proptosis, and an eyelid coloboma, and patient 261,321, with a larger deletion yet, is reported simply with abnormal scleral morphology. Thus, *SMO* haploinsufficiency does not clearly affect the human eye or brain, reflecting possible rescue by the remaining allele.

Murine *Smo* null (homozygous) mutants also do not demonstrate HPE, but they do have ocular defects that correlate with mispatterning of the DV axis, reminiscent of loss of the Gli2 or Otx2 effectors of Shh signaling. In these mutants, *Bmp4*, usually expressed in the dorsal portion of the developing eye, expands ventrally, accompanied by reduction in transcription of *Vax2* and *Pax2* transcription factors and subsequent impairment of ventral eye formation (Zhao et al. 2010). This antagonistic relationship between Bmp4 and Shh in patterning the DV axis of the ocular primordium has also been described in *Xenopus* (Sasagawa et al. 2002) and chick (Ohkubo et al. 2002; Kobayashi et al. 2010).

Shh-mediated signaling, by binding Ptch, frees up Smo for actual transduction. In vertebrates, this involves Smo modification by cholesterol and relocalization to the sensory organelle, the primary cilium (Fig. 1; reviewed by Briscoe and Thérond 2013; Byrne et al. 2018). This process remains incompletely understood to date. In *Drosophila*, activated Smo liberates the heterotrimeric G protein subunit, Gαi, decreasing levels of adenylate cyclase-mediated PKA and attenuating Ci cleavage to the repressor form (Ogden et al. 2008). However, Smo may also affect Gli-independent, Shh-induced cellular responses, in the light of conflicting data concerning the inhibitory effects of Gαi activation on PKA (Pandit and Ogden 2017). This may in part explain the chemotaxis displayed by some cranial NCC in the presence of Shh (Tolosa et al. 2016) as well as the very heterogeneous phenotypes in the presence of mosaic, constitutively activating mutations in *SMO*. Depending on the lineages targeted, these can cause Curry–Jones syndrome, which includes iris colobomas and microphthalmia (Twigg et al. 2016), while within the epidermis in postnatal somatic mutation, the same hotspot mutations are found in basal cell carcinomas (Xie et al. 1998).

### CDON, GAS1 and BOC

CDON, GAS1 and BOC are transmembrane proteins that act as co-receptors for SHH and are involved in the modulation of cellular response to SHH activity. Cdon complexes with many proteins, including Ptch1 but also Boc (“brother of CDON”), through its fibronectin domains. Missense substitutions in *CDON* induce HPE by continuing to bind SHH but not PTCH1, thereby sequestering and diminishing the perceived levels of SHH in target cells (Bae et al. 2011). These studies indicate a role for CDON in mammals as a positive modulator of the Hh pathway. However, *cdon* may also have a role as a negative modulator of the pathway. In zebrafish and chick embryos, unmutated Cdon has been shown to work as a decoy by sequestering, and thereby attenuating, Shh activity. Consistently, downregulation of *cdon* leads to an expansion of optic stalk fate at the expense of distal optic vesicle fates (Cardozo et al. 2014).

*BOC* has only recently been identified as a true HPE modifier gene in concert with *SHH* mutations (Hong et al. 2017). GAS1 is a GPI-linked co-receptor that also interacts directly with SHH in ways that remain biochemically unclear; missense heterozygous mutations are an uncommon cause of HPE along the entire phenotypic spectrum (Ribeiro et al. 2010; Pineda-Alvarez et al. 2012).

In mice homozygous null for *Gas1*, presenting a mild HPE midline phenotype, it has been shown that additional loss of Boc alleles plays a modifier role that demonstrates that many of the pleiotropic effects of Shh signaling are mediated by combinatorial and sometimes unique transduction through these receptors (Seppala et al. 2014). These may also be modulated by the non-specific *Lrp2*/megalin co-receptor. A recent review addresses this newly uncovered complexity in Shh signaling modulation (Xavier et al. 2016), although to date, no clear phenotype–genotype correlations have been drawn in animal models or human genetics between a particular receptor mutation subtype or combination, and the outcome with respect to HPE or ODA.

## Ciliopathies and Shh signal transduction

Cilia are specialized organelles present on nearly all cell types and which play a particularly important role in the organization and function of ocular tissues such as the RPE throughout life. They are also necessary for signal transduction by Shh in vertebrates (Briscoe and Thérond 2013). As such, many syndromic ciliopathies, such as Joubert syndrome and associated disorders, show disruption of processes in ocular formation that depend on intact signaling mediated by cilia (Khan et al. 2008; Valente et al. 2008). The many complex roles of cilia in ocular physiology and homeostasis are the subject of a complete and recent review in itself (May-Simera et al. 2017).

In short, Ptch binding of Shh leads to its replacement in the ciliary membrane by Smo (Rohatgi et al. 2007) and subsequent action on Gli subcellular localization (cf. Fig. 1). This mobilization is carried out specifically by the Sufu protein, in concert with kinesins and intraflagellar transport (IFT) proteins that are more generally critical for ciliary function. Although ancestral Hh signaling may not have required a primary cilium, transduction through ciliary Smo is also conserved in *Drosophila* olfactory neurons (Kuzhandaivel et al. 2014).

### Suppressor of fused (SUFU) is a key molecular chaperone of Gli effector TFs

SUFU is a molecular chaperone protein critical for the ciliary trafficking and processing of Gli proteins. To regulate its localization, it can be phosphorylated by protein kinase A (PKA) or glycogen synthase kinase 3 (GSK3). Mutations in *SUFU* are a rarer cause of BCN syndrome, along with mutations in *PTCH2* or the principal gene *PTCH1* (Johnson et al. 1996), compatible with a normal role in modulating and restraining the effects of SHH signaling. High-throughput sequencing in human patients suggests that variants in additional SHH pathway genes may also contribute to BCN syndrome phenotypes (Onodera et al. 2017). Ocular anomalies, a minor diagnostic criterion, may be under-reported but include congenital cataract, coloboma and glaucoma—while further emerging reports of somatic mosaicism in any of the BCN causative genes, may yet prove to be the cause of some apparently de novo ODA.

In the absence of SHH, SUFU constitutively sequesters GLI2/3 proteins at the base of ciliary microtubules. Translocation of this complex to the tips of cilia then promotes Gli TF cleavage into repressor forms, and Sufu has recently been shown to accompany these to the nucleus as far as the chromatin itself. In the presence of SHH, SUFU instead traffics GLI1/2/3 activator forms into the nucleus and the GLI2/3 repressor forms out (Zhang 2017).

### Examples of kinesins crucial for SHH signal transduction

The kinesins are a superfamily of molecular motors that hydrolyse ATP to translocate cargoes along the microtubules of primary cilia, towards the tip. Mutations affecting intraflagellar transport and kinesin proteins can cause ocular ciliopathies like retinitis pigmentosa (RP) by preventing the transport of proteins necessary for photoreceptor development, function and survival. They also can cause misregulation of Shh signal transduction by playing havoc with the trafficking of Gli TFs (reviewed in Bisgrove and Yost 2006).

Structural organization of the tip of cilia by the kinesin KIF7 (He et al. 2014) is critical for association with the small GTPase ARL3 and the GTPase-activating protein, Retinitis Pigmentosa 2 (RP2), mutations of which, as its name implies, cause an X-linked form of RP (Schwarz et al. 2017). Certain missense mutations in *KIF7* (Dafinger et al. 2011), and more infrequently, *ARL3* (Strom et al. 2016) and other proteins in this complex, as well as *SUFU* itself (De Mori et al. 2017), are all causes of Joubert syndrome. Early reports of this syndrome and the related but more severe Meckel syndrome, including fetal phenotyping, have shown multiple associations with coloboma and micro/anophthalmia (Delous et al. 2007; reviewed by; Valente et al. 2008; May-Simera et al. 2017).

However, even fetuses affected with the severe hydrolethalus syndrome, due to *KIF7* mutation, are much more frequently affected by brain malformations to the point of anencephaly, and by hypertelorism, than ODA, although one case was described with bilateral optic atrophy (Putoux et al. 2011). Indeed, the very *KIF7* mutations identified in these severe cases lead to upregulation of dozens of direct and indirect SHH signaling targets, through augmenting the ratio of activator to repressor forms of GLI3 protein (Putoux 2011). Similarly, *Kif3a* mutations in mouse NCC lead to complete loss of their cilia, increased proliferation and *Gli1* expression, and thereby phenocopy the effects of excessive Shh signaling on the forming face (Brugmann et al. 2010). This leads to hypertelorism and frontonasal dysplasia but have no major effects on eye formation before E12.5, by which time retinal ganglion cells are extending axons into the optic nerve, the corneal mesenchyme is in place, the lens is differentiating and the RPE beginning to pigment. Many other mutations in genes for ciliary proteins have a similar effect on craniofacial mesenchyme and are associated with hypertelorism but not with ODA as such.

### Ciliary shape and integrity determines Shh signaling efficacy

A number of recent studies in mice show a requirement for IFT proteins in controlling eye patterning through the modulation of Hh activity. For example, Ift172 is required for anterograde ciliary transport (toward the tip) while Ift122 is required for retrograde transport (toward the base). Mouse mutants in *Ift172* do not produce cilia, while loss of *Ift122* leads to bulbous cilia, where cargo accumulates at the tip of these structures (Burnett et al. 2017; Fig. 1). These mutants also show defective patterning of the optic primordium along the proximo-distal axis, suggesting perturbed Shh signaling.

Indeed, *Ift172*^−/−^ optic cups show expansion of *Pax2* and *Sox1* expression at the expense of *Pax6*, which is reduced to a small patch at the distal-most region of the primordium, indicating relative reduction in optic cup- and expansion of optic stalk-fated territories. Likewise, expression of the TFs *Mitf* and *Otx2*, markers of the RPE, abnormally extends into the inner layer of the optic cup, with a concomitant reduction in the NR-specific TF *Chx10*, indicating that loss of Ift-mediated Shh signaling leads to expansion of proximal RPE fate at the expense of the more distal NR fate. Curiously, *Ift122*^−/−^ optic cups show a more severe phenotype, with no invagination of the optic vesicle at all. *Pax2* and *Sox1* are dramatically expanded throughout the mutant optic vesicle, while *Pax6* expression is virtually absent, and neither RPE nor NR is specified at all. Thus, Ift122-negative optic primordia adopt a full optic stalk fate. The phenotypes observed in *Ift177* and *Ift122* mutant embryos suggest overactivation of the HH pathway under conditions in which cilia formation is compromised, correlated in phenotypes with the extent of stimulation. Astonishingly, the concomitant knockout of *Ift122* and *Gli2* can partially rescue optic cup patterning and morphogenesis (Burnett et al. 2017).

We can hypothesize that overexpressing PHS-causing truncated forms of Gli3-R would make for a fascinating demonstration that some ciliary production of Gli3-R is necessary to counter baseline Gli2 activity, perhaps even in the absence of Shh as facilitated by Foxc1 and perhaps other co-factors. Altogether, defective cilia formation in these IFT mutants seems to lead to an increased proportion of activating to repressor forms of Gli TFs, mimicking constitutive signaling from the Hh pathway, and thereby affecting proximo-distal cell identity in the optic vesicle and its derivatives.

Activating intracellular cAMP (as mimicked by phorbol esters) and thereby, protein kinase A (PKA) early in development to further the proteolytic processing of Gli repressor forms may also counter these effects (Lauth et al. 2007). Application to human malformations can only be speculative concerning the use of autologous induced pluripotent cells for tissue reconstruction. Accordingly, since the Ptch-expressing RPE depends on Shh signaling via *Otx2* and *Mitf* induction, augmenting cAMP prevents RPE cell proliferation (Hecquet et al. 2002; Fig. 2).

The mutation of a lipid-modifying enzyme encoded by the *INPP5E* gene induces a form of Joubert syndrome (Bielas et al. 2009). *INPP5E* is an evolutionarily divergent paralog of the X-linked *OCRL* gene, which encodes another inositol polyphosphate phosphatase whose mutation can cause either the oculocerebrorenal syndrome of Lowe or a more restricted renal tubulopathy (Zhang et al. 1995). Although *OCRL* haploinsufficiency is frequently associated with congenital cataracts and glaucoma, these do not overlap with the retinitis pigmentosa or other ocular signs sometimes found in Joubert patients. However, INPP5E has been shown to compartmentalize certain lipids in the ciliary membrane, thereby regulating the concentration of an inhibitory transmembrane receptor known as GPR161 that directly augments cAMP content in the cilia and, thereby, prevents Shh-dependent GLI1 activity (Chávez et al. 2015). *INPP5E*-mutated primary cilia are shorter (Luo et al. 2012), indirectly accumulate too much GPR161 and constitutively repress at least some aspects of SHH signaling. In contrast, *OCRL*-mutated primary cilia can vary in length, depending on the model system studied, and are deficient in trafficking from endosomes to the ciliary membrane, thereby potentially affecting the levels of the non-specific SHH co-receptor, LRP2, or otherwise interfering with other signaling processes mediated by the primary cilium (Rbaibi et al. 2012).

Recently published work demonstrates that *Ccrk* (cell cycle-related kinase)-null mouse mutants show abnormal, shorter cilia, and display a number of corresponding developmental defects, amongst which microphthalmia due to fully penetrant coloboma and frequent loss of the lens primordium (Lupu et al. 2018). A result of disrupting Shh-dependent proximo-distal patterning at optic cup stages, malformations and effects on target gene transcription induced by mutant Ccrk confirm the hypothesis that different Shh activity readout levels lead to different ocular fates. Ccrk is required in regions of high levels of HH activity (proximal) to promote pathway activation, but it is simultaneously required in regions of low HH activity (distal) to maintain the pathway in a repressed state (Fig. 1). In *Ccrk* knockouts, the Gli1 expression domain of the distal optic vesicle is expanded and *Mitf* is ectopically expressed within the optic cup, promoting a RPE fate. Furthermore, *Ccrk* mutation rescues the effect of *Smo* mutants, in which *Pax6* is expressed throughout the entire optic vesicle following loss of Shh signaling, demonstrating that Ccrk acts downstream of Smo. The analysis of both *Ccrk*^−/−^;*Gli2*^−/−^ double mutants showed compensation for the excess distal Shh signaling activity mediated by the loss of *Ccrk* in terms of ocular vesicle morphology, although at the expense of ventral and midline tissues with normally highest exposure to Shh. Likewise, *Ccrk*^−/−^;*Gli3*^−/−^ double mutants show exacerbation of the distal RPE phenotype at the expense of neural retina fate, and total loss of lens specification (Lupu et al. 2018), supporting the idea that such effects result from loss of the Gli3-R TF form and expansion of midline fates at the expense of distal tissues.

## Conclusions

The vertebrate eye is a highly complex structure whose assembly is coordinated over time from four distinct sources of embryological cells. Hedgehog signaling plays a role from the very first stages of specification of the neural epithelium, to determining the eye fields, to the maintenance of healthy functional ocular tissues in the adult organism. Over the last 25 years, the number of molecular participants in this pathway has grown exponentially, as have demonstrations that changes in their activity or availability cause human ocular disease. The primary cilium has been shown to be the principal organelle necessary for hedgehog signal reception and integration. The presence of the cilium is cell cycle-dependent, implying that Hh activity may also be cell cycle-dependent, adding one more level of complexity to the modulation of cellular functions by this signaling pathway. A “rheostat” model, first used to describe the gradation in signaling levels of the TF Mitf and in opposition to a binary on–off switch model (Carreira et al. 2006), best describes the continuous transcriptional integration of Gli and other TF at the level of Shh target gene chromatin. Perhaps we should consider the “trimpot” (trimmer potentiometer) as a more apt metaphor for the variable transcriptional effects of Shh signaling, as the adjustment periods for reactivity to thresholds are limited during development. These fascinating mechanisms will continue to surprise and inform our understanding of human pathology, as additional biological circuitry is explored and brought to light in the future.
